# Underinsurance Among Children With Special Health Care Needs in the United States

**DOI:** 10.1001/jamanetworkopen.2023.48890

**Published:** 2023-12-26

**Authors:** Asiya Validova, Douglas Strane, Meredith Matone, Xi Wang, Rebecka Rosenquist, Xianqun Luan, David Rubin

**Affiliations:** 1 PolicyLab, Children’s Hospital of Philadelphia, Philadelphia, Pennsylvania; 2 Department of Pediatrics, Perelman School of Medicine at the University of Pennsylvania, Philadelphia; 3 Leonard Davis Institute of Health Economics at University of Pennsylvania, Philadelphia

## Abstract

**Importance:**

A rise in pediatric underinsurance during the last decade among households with children with special health care needs (CSHCN) requires a better understanding of which households, by health care burden or income level, have been most impacted.

**Objective:**

To examine the prevalence of underinsurance across categories of child medical complexity and the variation in underinsurance within these categories across different levels of household income.

**Design, Setting, and Participants:**

This cross-sectional study used data from the National Survey of Children’s Health and included 218 621 US children from 2016 to 2021. All children included did not reside in any type of institution (eg, correctional institutions, juvenile facilities, orphanages, long-term care facilities). Data were analyzed from January 2016 to December 2021.

**Exposures:**

The primary exposure is a categorization of child health care needs constructed using parent-reported child physical and behavioral health conditions, as well as the presence of functional limitations.

**Main Outcomes and Measures:**

The primary outcome variable is underinsurance, defined as absence of consistent or adequate health insurance. Models were adjusted for demographic and socioeconomic characteristics and stratified by household income. Multivariate logistic regression analysis of pooled cross-sectional survey data across multiple years (2016 to 2021) adjusted for complex survey design (weights).

**Results:**

In a total sample of 218 621 children who were not in institutions and were aged 0 to 17 years from 2016 to 2021 (105 478 [48.9%] female; 113 143 [51.1%] male; 13 571 [13.0%] non-Hispanic Black children; 149 706 [51.2%] non-Hispanic White children), underinsurance prevalence was higher among the children who had complex physical conditions (3316 [37.0%]), mental or behavioral conditions (5432 [38.1%]), or complex physical conditions and functional limitations (1407 [40.7%]) or mental or behavioral conditions with limitations (3442 [41.1%]), compared with healthy children (ie, children without special health care needs or limitations) (52 429 [31.2%]). The association between underinsurance and complexity of child health care needs varied by household income. In households earning 200% to 399% federal poverty level (FPL), underinsurance was associated with children having complex physical conditions and limitations (OR, 2.74; 95% CI, 2.13-3.51) and mental or behavioral conditions and limitations (OR, 2.21; 95% CI, 1.87-2.62), compared with healthy children. In households earning 400% or more above FPL, children’s mental or behavioral conditions and limitations were associated with underinsurance (OR, 3.31; 95% CI, 2.82-3.88) compared with healthy children.

**Conclusions and relevance:**

In this cross-sectional study, the odds of being underinsured were not uniform among CSHCN. Both medical complexity and daily functional limitations led to increased odds of being underinsured. The concentration of underinsurance among middle-income households underpinned the challenge of health care financing for families of CSHCN whose incomes surpassed eligibility thresholds for dependent Medicaid insurance.

## Key Points

**Question:**

Is medical complexity of child physical or behavioral health care needs associated with the risk of underinsurance among children across different household income levels?

**Findings:**

In this cross-sectional study of 218 621 US children from 2016 to 2021, the prevalence of underinsurance was higher among children with complex physical and/or behavioral health care needs compared with healthy children (ie, children without special health care needs). For most child health care needs categories, underinsurance was highest among families with incomes between 200% and 399% of federal poverty level.

**Meaning:**

These findings suggest that chronic medical or behavioral health issues in children who have functional limitations compound the odds of being underinsured, particularly for children from middle-income households.

## Teaser

This cross-sectional study examines the prevalence of underinsurance across categories of child medical complexity and the variation in underinsurance within these categories across levels of household income.

## Introduction

Health insurance allows children to access health care services necessary to develop and thrive while protecting their families from catastrophic medical expenditures and the high cost of health care. Children who are underinsured have inadequate health insurance coverage in the form of prohibitive out-of-pocket expenditures, narrow clinician networks, an insufficient package of benefits, or inconsistent enrollment. Childhood underinsurance is, in turn, associated with lower reported quality of care, forgone care, and unmet health needs.^1,2,3^ Though the proportion of children with health insurance has reached historic highs in the past decade, this trend has coincided with a rise in the prevalence of pediatric underinsurance.^4,5,6^ These changes to the adequacy of children’s health insurance coverage largely reflect changes in the commercial or employer-sponsored insurance market, which covers half of children in the United States. Families enrolled in such plans have experienced substantial growth in premiums and deductibles throughout the past decade.^5^

The implications of underinsurance may disproportionately affect the families of children with special health care needs (CSHCN), who require more frequent access to health care and disease management services than their peers without chronic conditions. CSHCN, who represented 19.4% of children in the United States in 2020, generated a large percentage of health care expenses for children.^7^ They are also at elevated risk of hospitalizations, emergency department visits, and unplanned doctor’s visits.^8,9^ In 2019, an estimated 40% of CSHCN in the United States were underinsured compared with 32% of healthy children (ie, children without special health care needs or limitations) who were underinsured.^6^ Owing to their ongoing need for clinical services, CSHCN are particularly vulnerable to potential health care access disruptions that may arise due to cost barriers related to underinsurance.

CSHCN with widely varying needs have variable experiences with health insurance adequacy due to differences in the type, predictability, and frequency of care they require.^10^ For example, the care needs of a child with a complex physical health condition like congenital heart disease can be intensive but quite different than the intensive needs—particularly for daily care—of a child with autism spectrum disorder.^11,12^ While previous research has indicated that CSHCN do not experience a uniform risk of underinsurance, there is a need to more rigorously examine the differential risk of underinsurance across types and severity of pediatric conditions. Furthermore, we might expect financial strain associated with health care costs to be experienced more acutely by families of CSHCN with limited financial means, particularly if their children are ineligible for Medicaid or the Children’s Health Insurance Programs (CHIP).^13^ Children enrolled in public insurance are less likely to be underinsured than those with private insurance coverage and CSHCN with public coverage report lower cost-sharing.^14^ To fully understand CSHCN’s risk of underinsurance, it is important to examine their level of medical complexity alongside their family’s income level.

In an era of low rates of uninsurance but rising costs for private health insurance and health care services, there is a need to understand the adequacy of health insurance coverage for children who use the most health care resources. Few studies have explored pediatric underinsurance while also considering the highly variable needs of CSHCN, particularly in recent years.^10,15,16^ In this nationally representative study of children in the United States, we analyzed data from the National Survey of Children’s Health (NSCH) from 2016 to 2021 to examine the prevalence of underinsurance across discrete categories that reflect the nature and medical complexity of children’s health conditions, as well as the presence of functional limitations. We additionally examine how household income is associated with underinsurance across these categories of child health needs.

## Methods

This cross-sectional study followed the Strengthening the Reporting of Observational Studies in Epidemiology (STROBE) reporting guideline. The study used publicly available data and was therefore determined to be exempt from formal review and informed consent by the institutional review board of Children’s Hospital of Philadelphia.

Data used for this analysis came from the National Survey of Children’s Health (NSCH), which provides national and state-level estimates on key indicators of children’s physical and emotional health and well-being, health care access, and utilization, as well as presence and impact of special health care needs. NSCH uses 2-phase data collection approach with an initial household screener followed by a topical questionnaire completed by a parent or caregiver for 1 randomly selected child from the household. Data are weighted to be a representative sample of the US population of children aged 0 to 17 years who are not in institutions (eg, correctional institutions, juvenile facilities, orphanages, long-term care facilities).^17^

We combined data across multiple years (ie, 2016 to 2021) to increase the analytical sample. Combined weights were used for multiyear analysis. Our total sample included 218 621 children.

### Measures

#### Dependent Variable

This study examined underinsurance as the primary outcome variable to understand the adequacy of insurance for CSHCN. NSCH uses a set of items to operationalize continuous and adequate insurance. To qualify for this measure the selected child must meet 2 criteria: (1) have continuous insurance in the past 12 months and (2) have current insurance that is adequate for the child’s health care needs. In turn, several survey questions address the adequacy of the child’s health insurance coverage. To meet insurance adequacy criteria the following insurance requirements must be met, as per a caregiver’s response in the survey: (1) benefits usually or always meet child’s needs (vs sometimes or never), and (2) the insurance usually or always allows the child to see needed health care practitioners (vs sometimes or never), and (3) the insurance either has no out-of-pocket expenses or out-of-pocket expenses are usually or always reasonable (vs sometimes or never). Children with positive responses to those measures were categorized as having continuous and adequate insurance; those with other nonmissing responses were considered underinsured.

#### Primary Exposure

The NSCH uses responses to CSHCN screener questions to classify children into 2 broad categories: children with special health care needs (SHCN) and non-SHCN.^18^ The survey identifies SHCN based on health consequences that child experiences due to ongoing health condition rather than relying on specific diagnoses.

Using specific questions about the children’s needs, we stratified the SHCN category to address medical complexity across domains of physical and behavioral health conditions, as well as the presence of functional limitations resulting from their health conditions. This allowed us to distinguish between children with less complex needs that can be managed solely by means of prescribed medication, all the way up to children with significant limitations that required more intensive daily care and specialty program involvement.

The following survey questions were used to define a child’s health care needs status: (1) use of prescription medication; (2) elevated need of medical care or need of specialized therapy; (3) need for mental, behavioral, or developmental health treatment; and (4) functional limitations because of their condition. Based on these items, we created 6 mutually exclusive categories of child health care needs: (1) healthy children; (2) children with ongoing medication needs (ie, conditions that are addressed solely by prescribed medications); (3) children with complex physical conditions but no limitations; (4) children with mental or behavioral conditions but no limitations; (5) children with complex physical conditions and limitations; and (6) children with mental or behavioral conditions and limitations. Additionally, we created diagnostic profiles to examine the most prevalent diagnoses for each category of children (eTable 1 in Supplement 1).

Healthy children were used as a reference group for the analysis. When children had both service needs for physical and behavioral health care, the default was to include them among children with behavioral health needs due to sample size limitations that limited our ability to evaluate children in dual categories separately.

#### Other Independent Variables

We considered other demographic and socioeconomic characteristics from NSCH that might have confounded an association between the complexity of child health care needs and underinsurance. These variables included age, sex, race or ethnicity, and child immigrant status. Family socioeconomic factors in the analysis included parental educational attainment, household income, and type of health insurance coverage. Race and ethnicity were distinguished between 4 categories: Hispanic, non-Hispanic Black, non-Hispanic White (reference category), and other racial or ethnic group. Questions about a child’s Hispanic ethnicity were asked in the initial screening questionnaire. Non-Hispanic children reporting 1 race category of American Indian or Alaska Native, Asian, Native Hawaiian or Other Pacific Islander, and more than 1 race were grouped as other. The full description of variables’ construction can be found in the eAppendix of Supplement 1.

### Statistical Analysis

Characteristics of the population are described across health care needs categories and underinsurance status. The results are displayed as unweighted numbers and weighted percentages in accordance with the NSCH survey weighting methods.^17^

Multivariate logistic regression analysis was conducted through a sequential model-building approach. First, we examined the association between a child’s health care needs and underinsurance using unadjusted logistic regression model, to which we then added demographic and socioeconomic variables as potential confounders. We then considered whether the association between child health care needs and underinsurance was modified by household income level. With an α error <.05 for the interaction between child health care needs and household income, we conducted logistic regression analysis stratifying our sample by household income and performed postestimation to generate adjusted estimated probabilities of underinsurance (with confidence intervals) across categories of income and health care needs (Figure).

**Figure.**
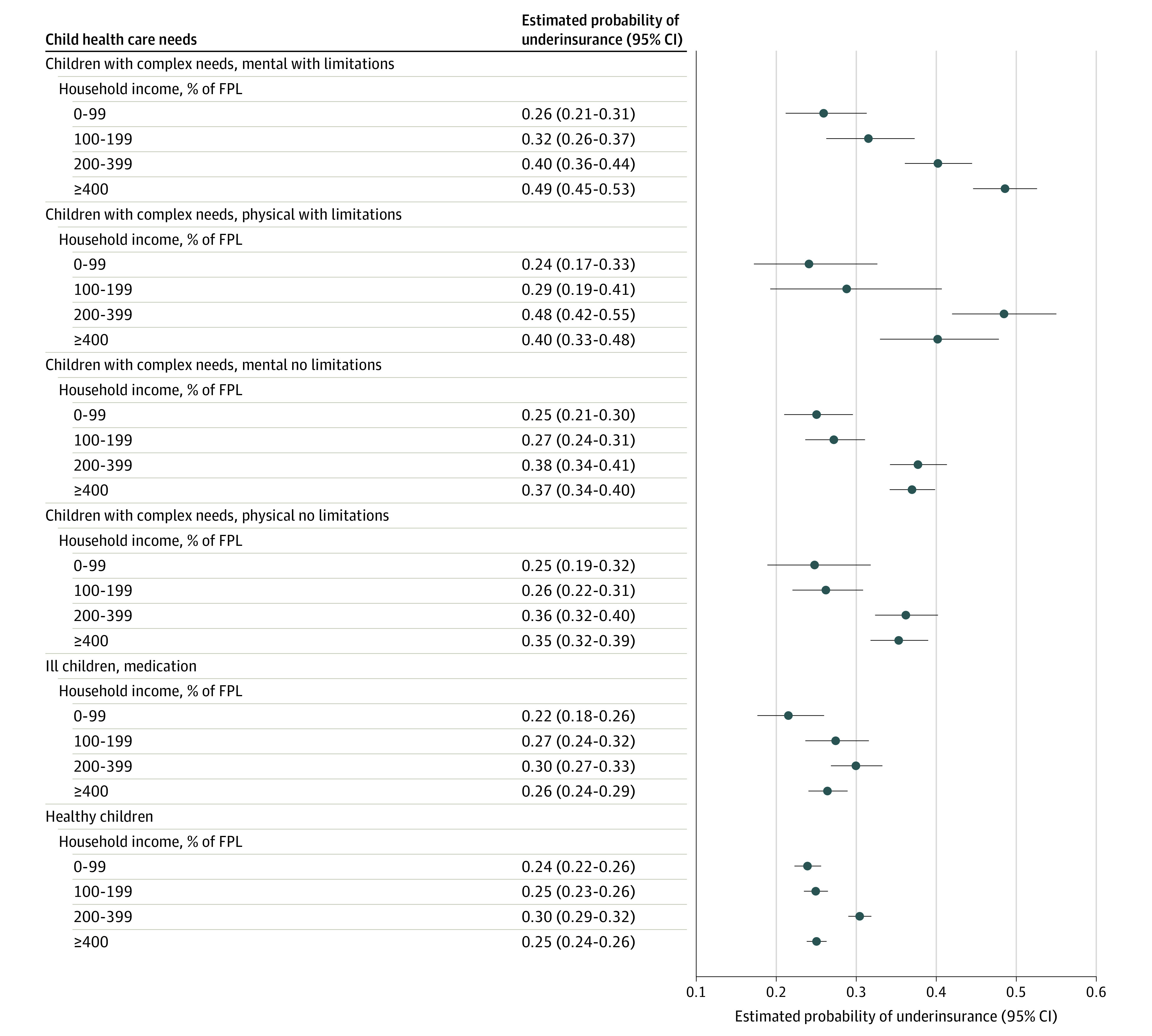
Estimated Probability of Underinsurance Among Children in the United States by Child Health Care Needs Categories and Household Income, National Survey of Children’s Health, 2016 to 2021 The figure used data from the National Survey of Children’s Health, 2016 to 2021. Dots indicate adjusted estimated probabilities of underinsurance and lines indicate 95% CIs. Estimated probability of underinsurance was calculated based on the model adjusted for a set of control variables including child’s sex, age, race or ethnicity, nativity, parental education, and year. No. (unweighted) = 217 926. FPL indicates federal poverty level.

Analyses were conducted in R version 4.3.1 (R Project for Statistical Computing) and accounted for complex survey design to produce estimates representative of US children aged 0 to 17 years who were not in institutions. Data were analyzed from January 2016 to December 2021. Statistical significance was set at *P* < .05, and tests were 2-sided.

## Results

### Descriptive Statistics

This study included a total sample of 218 621 children aged 0 to 17 years from 2016 to 2021 who were not in institutions (105 478 [48.9%] female; 113 143 [51.1%] male; 13 571 [13.0%] non-Hispanic Black children; 149 706 [51.2%] non-Hispanic White children). Descriptive statistics for the analytical sample are presented in Table 1 and eTable 2 in Supplement 1. Between 2016 and 2021, 70 431 children (32.3%) were underinsured. In total, 47 057 children (17.9%) had SHCN, which included 13 654 (4.8%) with prescription needs only; 8025 (3.0%) with physical health needs but no limitations; 14 265 (5.4%) with mental or behavioral conditions but no limitations; 3091 (1.3%) with physical health needs and limitations; and 8022 (3.4%) with mental or behavioral conditions and limitations.

**Table 1.** Characteristics and Underinsurance Status of Children in the United States, National Survey of Children’s Health, 2016-2021

Characteristic	Full sample, No. (column weighted %)^a^^,^^b^	Underinsured, No. (row weighted %)^a^^,^^c^
No. (unweighted)	218 621	70 431 (32.3)^d^
Children’s health care needs category^e^		
Healthy children	170 869 (82.1)	52 429 (31.2)
Children with ongoing medication needs	13 654 (4.8)	4405 (31.9)
Children with complex physical conditions but no limitations	8025 (3.0)	3316 (37.0)
Children with mental or behavioral conditions but no limitations	14 265 (5.4)	5432 (38.1)
Children with complex physical conditions and limitations	3091 (1.3)	1407 (40.7)
Children with mental or behavioral conditions and limitations	8022 (3.4)	3442 (41.1)
Underinsurance^e^		
Insured	147 495 (67.7)	NA
Underinsured	70 431 (32.3)	NA
Age group		
0-5 years	68 391 (32.3)	19 774 (28.9)
6-11 years	65 016 (33.6)	20 758 (32.0)
12-17 years	85 214 (34.1)	29 899 (35.7)
Sex		
Female	105 478 (48.9)	33 925 (32.7)
Male	113 143 (51.1)	36 506 (31.9)
Race or ethnicity		
Non-Hispanic Black	13 571 (13.0)	3783 (27.9)
Hispanic	26 646 (24.9)	8970 (34.5)
Non-Hispanic White	149 706 (51.2)	48 778 (32.5)
Other^f^	28 698 (10.9)	8900 (31.3)
Nativity		
First generation immigrants	3652 (2.9)	1557 (46.8)
Second generation immigrants	34 246 (22.9)	11 422 (33.3)
≥Third generation	169 906 (67.2)	54 608 (31.8)
Other or unknown	10 817 (6.9)	2844 (26.9)
Household income, % of FPL		
0-99	24 825 (19.1)	6943 (28.7)
100-199	35 071 (21.4)	10 658 (30.6)
200-399	67 383 (28.1)	24 606 (36.7)
≥400	91 342 (31.3)	28 224 (31.6)
Parental education		
Less than high school	5154 (8.9)	1859 (36.7)
High school	27 841 (19.3)	8450 (29.5)
Some college	49 222 (21.5)	16 120 (31.7)
College or more	136 404 (50.3)	44 002 (32.8)
Type of health insurance coverage^g^		
Public only	44 695 (30.5)	6465 (15.0)
Private only	155 644 (58.3)	51 853 (33.7)
Private and public	8394 (4.6)	2566 (31.0)
Not insured	9401	9401 (100.0)

Abbreviations: FPL, federal poverty level; NA, not available.

^a^ No. is the number of observations (unweighted), weighted percentage is calculated adjusting for weights and survey design.

^b^ Weighted percentage of the column.

^c^ Weighted percentage of the row, which represents the percentage of underinsured across the demographic and socioeconomic variables.

^d^ Percentage of the full sample.

^e^ The variables children’s health care needs and underinsurance categories had 695 missing observations.

^f^ Other includes Non-Hispanic children reporting 1 race category of American Indian or Alaska Native, Asian, Native Hawaiian or Other Pacific Islander, and children who reported more than 1 race.

^g^ This variable had 487 missing observations.

Underinsurance prevalence varied across the child health care needs categories (Table 1). Prevalence of underinsurance was higher among children who had physical health conditions (3316 [37.0%]) or mental or behavioral health conditions (5432 [38.1%]) compared with healthy children (52 429 [31.2%]). Among children with mental or behavioral (3442 [41.1%]) or complex physical conditions and functional limitations (1407 [40.7%]) were underinsured.

Underinsurance also varied across demographic characteristics. Underinsurance was highest in the 12 to 17 years age group (29 899 [36%] vs 19 774 [28.9%] in the 0 to 5 years age group and 20 758 [32.0%] in the 6 to 11 years age group); among Hispanic children (8970 [34.5%] vs 3783 [27.9.%] among non-Hispanic Black children and 48 778 [32.5%] among non-Hispanic White children); and among children of parents without education beyond high school (1859 [36.7%] for less than a high school education vs 44 002 [32.8%] for those a college degree or higher).

The prevalence of underinsurance varied across income levels, with the highest prevalence of underinsurance among children in households earning 200% to 399% of the federal poverty level (FPL), 24 606 (36.7%) of whom were underinsured. In contrast, 6943 (28.7%) of children in households earning 0% to 99% of the FPL were underinsured, as well as 10 658 (30.6%) in households earning 100% to 199% of the FPL, and 28 224 (31.6%) in households earning 400% or more of the FPL. Finally, there was a large variation in underinsurance across types of insurance coverage. The prevalence of underinsurance among children with private or a combination of public and private coverage was nearly twice as high as among children covered by public health insurance only (2566 [31.0%] for public and private health insurance to 51 853 [33.7%] for private health insurance coverage only vs 6465 [15.0%]) (Table 1). eTable 2 in Supplement 1 further disaggregates sociodemographic characteristics of children across the level of health care needs in our sample.

### Adjusted Associations With Underinsurance

The unadjusted logistic regression model results report the significant positive association between child health care needs and underinsurance (Table 2). Logistic regression models adjusted for demographic and socioeconomic variables further demonstrate that category of child health care needs is significantly associated with underinsurance (Table 2). Children with complex physical and mental or behavioral needs and no limitations compared with their healthy counterparts had 1.31 (95% CI, 1.18-1.45) and 1.35 (95% CI, 1.25-1.47) times the odds of being underinsured, respectively. The strongest association was found among children with complex needs (due to both physical and/or mental conditions) who also had functional limitations (OR, 1.57; 95% CI, 1.30-1.90 among children with physical conditions and limitations; OR, 1.62; 95% CI, 1.45-1.80 among children with mental or behavioral conditions and limitations). Income level was also independently associated with underinsurance; among income brackets, the strongest association was found among those with incomes between 200% to 399% of the FPL (OR, 1.51; 95% CI, 1.40-1.63).

**Table 2.** Adjusted Odds of Underinsurance Among Children in the United States, National Survey of Children’s Health 2016-2021^a^

Variables	OR (95% CI)		
	Model 1 (n = 217 926)^b^	Model 2 (n = 217 926)^c^	Model 3 (n = 217 926)^d^
Children’s health care needs category (reference, healthy children)			
Children with ongoing medication needs	1.03 (0.95-1.12)	1.03 (0.95-1.11)	0.89 (0.69-1.14)
Children with complex physical conditions but no limitations	1.29 (1.17-1.43)	1.31 (1.18-1.45)	1.06 (0.75-1.49)
Children with mental or behavioral conditions but no limitations	1.36 (1.25-1.47)	1.35 (1.25-1.47)	1.07 (0.85-1.35)
Children with complex physical conditions and limitations	1.51 (1.25-1.82)	1.57 (1.30-1.90)	1.01 (0.66-1.54)
Children with mental or behavioral conditions and limitations	1.53 (1.38-1.70)	1.62 (1.45-1.80)	1.12 (0.86-1.47)
Demographic controls			
Sex (reference, male)			
Female	NA	1.05 (1.01-1.10)	1.05 (1.01-1.10)
Age group (reference, 0-5)			
6-11	NA	1.12 (1.06-1.18)	1.12 (1.06-1.18)
12-17	NA	1.29 (1.22-1.36)	1.29 (1.22-1.35)
Race or ethnicity (reference, non-Hispanic White)			
Hispanic	NA	1.06 (0.99-1.14)	1.06 (0.99-1.13)
Non-Hispanic Black	NA	0.83 (0.77-0.89)	0.83 (0.78-0.90)
Other^e^	NA	0.91 (0.85-0.97)	0.91 (0.86-0.97)
Nativity (reference, ≥ third generation)			
First generation immigrants	NA	1.94 (1.69-2.23)	1.92 (1.66-2.21)
Second generation immigrants	NA	1.07 (1.00-1.14)	1.06 (1.00-1.14)
Other	NA	0.82 (0.74-0.91)	0.82 (0.74-0.91)
Socioeconomic variables			
Parental education (reference, less than high school)			
High school	NA	0.74 (0.66-0.84)	0.74 (0.66-0.84)
Some college	NA	0.78 (0.70,0.88)	0.79 (0.70-0.89)
College or higher	NA	0.80 (0.71-0.90)	0.80 (0.72-0.90)
Parental income (reference, 0%-99% FPL), % FPL			
100-199	NA	1.11 (1.03-1.21)	1.08 (0.99-1.18)
200-399	NA	1.51 (1.40-1.63)	1.43 (1.31-1.55)
≥400	NA	1.20 (1.11-1.29)	1.08 (1.00-1.18)
Year (reference, 2016)			
2017	NA	1.11 (1.03-1.20)	1.11 (1.03-1.20)
2018	NA	1.09 (1.02-1.16)	1.09 (1.01-1.16)
2019	NA	1.17 (1.09-1.25)	1.17 (1.09-1.25)
2020	NA	1.07 (1.00-1.14)	1.07 (1.00,1.14)
2021	NA	1.01 (0.95-1.08)	1.01 (0.95-1.08)
Child health care needs category × parental income		NA	NA
Children with ongoing medication needs × 100%-199% FPL	NA	NA	1.28 (0.93-1.76)
Children with ongoing medication needs × 200%-399% FPL	NA	NA	1.10 (0.83-1.48)
Children with ongoing medication needs × 400% FPL or greater	NA	NA	1.21 (0.92-1.59)
Children with complex physical conditions but no limitations × 100%-199% FPL	NA	NA	1.02 (0.67-1.54)
Children with complex physical conditions but no limitations × 200%-399% FPL	NA	NA	1.23 (0.84-1.81)
Children with complex physical conditions but no limitations × 400% FPL or greater	NA	NA	1.55 (1.06-2.25)
Children with mental or behavioral conditions but no limitations × 100%-199% FPL	NA	NA	1.05 (0.78-1.42)
Children with mental or behavioral conditions but no limitations × 200%-399% FPL	NA	NA	1.30 (0.99-1.71)
Children with mental or behavioral conditions but no limitations × 400% FPL or greater	NA	NA	1.65 (1.28-2.13)
Children with complex physical conditions and limitations × 100%-199% FPL	NA	NA	1.22 (0.62-2.41)
Children with complex physical conditions and limitations × 200%-399% FPL	NA	NA	2.12 (1.30-3.49)
Children with complex physical conditions and limitations × 400% FPL or greater	NA	NA	2.00 (1.19-3.37)
Children with mental or behavioral conditions and limitations × 100%-199% FPL	NA	NA	1.24 (0.86-1.80)
Children with mental or behavioral conditions and limitations × 200%-399% FPL	NA	NA	1.38 (1.00-1.90)
Children with mental or behavioral conditions and limitations × 400% FPL or greater	NA	NA	2.52 (1.85-3.44)
Constant	0.45 (0.44-0.47)	0.37 (0.32-0.42)	0.39 (0.35-0.44)

Abbreviations: FPL, federal poverty level; NA, not available; OR, odds ratio.

^a^ Models were adjusted for complex survey designs (weights).

^b^ Model 1 is unadjusted model.

^c^ Model 2 has been adjusted for demographic and socioeconomic variables.

^d^ Model 3 is an adjusted model which includes interaction between child health care needs and household income level.

^e^ Other included Non-Hispanic children reporting 1 race category of American Indian or Alaska Native, Asian, Native Hawaiian or Other Pacific Islander, and children who reported more than 1 race.

Through postestimation of estimated probabilities of underinsurance, we found that the estimated probability of being underinsured was substantially higher for the categories of children with complex physical needs and children with mental or behavioral needs than for healthy children and children with ongoing medication needs (Figure). The estimated probability of underinsurance among children with complex physical needs and functional limitations was 0.48 (95% CI, 0.42-0.55) among those with income 200% to 399% of the FPL and 0.40 (95% CI, 0.33-0.48) among those with income 400% or more of the FPL. Among children with mental or behavioral conditions and functional limitations, however, those from households with income 400% or more of the FPL had an estimated probability of 0.49 (95% CI, 0.45-0.53) of being underinsured compared with 0.40 (95% CI, 0.36-0.44) among those with incomes 200% to 399% FPL (Figure).The results of the logistic models stratified by household income presented in Table 3 show that the strength of the association between underinsurance and category of child health care needs varied by household income. In households with income between 200% to 399% FPL, children’s complex physical conditions and limitations (OR, 2.74; 95% CI, 2.13-3.51) and complex mental or behavioral conditions with limitations (OR, 2.21; 95% CI, 1.87-2.62) were associated with underinsurance relative to healthy children. In households with an income level of 400% or more of the FPL, children with complex mental or behavioral conditions and limitations representing the group most vulnerable to underinsurance, with 3.31 (95% CI, 2.82-3.88) times the odds of being underinsured compared with healthy children. Finally, children covered by private insurance were significantly more likely to be underinsured than those with public insurance across all income level groups; this association was more pronounced among households earning 0% to 99% and 100% to 199% of the FPL.

**Table 3.** Adjusted Odds of Underinsurance Among Children in the United States, Stratified by Household Income Group, National Survey of Children's Health 2016-2021^a^

Variables	OR (95% CI)			
	0%-99% FPL (n = 22 437)	100%-199% FPL (n = 32 533)	200%-399% FPL (n = 64 130)	≥400% FPL (n = 88 938)
Children’s health care needs category (reference, healthy children)				
Children with ongoing medication needs	1.23 (0.92-1.65)	1.37 (1.11-1.69)	1.17 (1.01-1.37)	1.16 (1.03-1.31)
Children with complex physical conditions and no limitations	1.78 (1.13-2.82)	1.49 (1.16-1.92)	1.60 (1.36-1.88)	1.69 (1.47-1.95)
Children with mental conditions and no limitations	1.72 (1.33-2.23)	1.68 (1.36-2.08)	1.86 (1.58-2.18)	2.01 (1.80-2.25)
Children with complex physical conditions and limitations	1.64 (1.06-2.55)	1.80 (0.99-3.29)	2.74 (2.13-3.51)	1.97 (1.49-2.61)
Children with mental conditions and limitations	2.09 (1.56-2.80)	2.36 (1.73-3.22)	2.21 (1.87-2.62)	3.31 (2.82-3.88)
Type of health insurance coverage (reference, public only)				
Private only	4.29 (3.62-5.08)	3.38 (2.96-3.86)	3.09 (2.70-3.55)	2.55 (2.05-3.17)
Private and public	2.60 (1.99-3.39)	2.22 (1.77-2.77)	2.19 (1.77-2.72)	2.29 (1.65-3.18)
Constant	0.11 (0.08-0.14)	0.10 (1.07-1.14)	0.15 (0.10-0.22)	0.09 (0.05-1.16)

Abbreviations: FPL, federal poverty level; OR, odds ratio.

^a^ Model was adjusted for a set of control variables including child’s sex, age, race or ethnicity, nativity, parental education, type of health insurance coverage, and year. ORs are presented only for child health care needs category and type of health insurance coverage variables as key predictors in the model. A full table with all the covariates is available upon request.

For the sensitivity analysis, we conducted a modified Poisson regression analysis and reported risk ratios for underinsurance (eTable 3 in Supplement 1). Our results were robust to model specifications.

## Discussion

The findings of our study suggest that CSHCN not only experienced underinsurance at higher levels than healthy children but that CSHCN were increasingly likely to be underinsured as their level of need and medical complexity increased. CSHCN in households earning 200% to 399% of the FPL were most likely to be underinsured overall; nearly half of children in this income group with complex physical conditions and limitations were underinsured. This concentration of underinsurance among middle-income families reveals a significant challenge of health care financing for families.

While often conceptualized in the literature as a uniform group, CSHCN are highly heterogenous, with health conditions that are diverse in complexity and daily needs that may require different intensities of care and supervision.^9^ Children with mental or behavioral conditions and children with physical health conditions require different types of specialty care and supportive services for which reimbursement is dependent on health insurance benefit packages (eg, for behavioral health services or durable medical equipment) that are highly variable by insurance plan.^19^ Out-of-pocket costs for services essential to CSHCN also vary greatly depending on whether a child is covered by public or commercial insurance, particularly commercial plans with high deductibles and coinsurance.^20,21^ By examining the prevalence of underinsurance across categories of child health care needs, we identified which groups of children face increased prevalence of underinsurance compared with their peers and how much that varied by household income or type of insurance coverage.

Our findings indicate that children in households earning 200% to 399% of FPL are more likely to be underinsured across all categories of child health needs, except children with mental or behavioral conditions and functional limitations, among whom those in households earning 400% or more of the FPL were most likely to be underinsured. These findings underscore the unique challenge of underinsurance for middle-income families caring for CSHCN. Children with complex physical conditions or limitations or mental or behavioral conditions require frequent specialty care visits, receive more medications, and are at greater risk of ED visits and hospitalization than other children.^12,22,23^ Children with mental or behavioral conditions and limitations—particularly those with intellectual disabilities—have a significant need for daily care and services from young childhood through adolescence.^19^ Our findings reveal a structural gap in access to state Medicaid and CHIP programs for families who earn too much to qualify for public health insurance and who do not qualify for Medicaid through a disability pathway but who are vulnerable to the costs of accessing necessary health care for their child. While our study examined a national-level cohort, the high prevalence of underinsurance among CSHCN from middle-income households is likely reflective of state-level variation in the 2 main criteria used to determine children’s eligibility for Medicaid coverage: household income and medical condition. Along with eligibility criteria, the type of coverage (ie, fee-for-service or managed care) provided to CSHCN enrolled in Medicaid varies across state lines.^24^ Compared with their peers enrolled in private insurance, CSHCN enrolled in public insurance have fewer out-of-pocket costs for medically necessary care, and parents are more likely to report that their health insurance always meets their children’s needs.^20^ As high cost-sharing for private health insurance coverage becomes more common, families of middle-income CSHCN without access to public insurance coverage in their state are left with a few options for comprehensive, affordable health insurance coverage for their child’s ongoing health care needs.^25,26^

### Limitations

This study has limitations. First, our CSHCN categories were based on categories defined by NSCH. Our categorization reflects the health consequences and medical complexity of a child’s condition rather than their diagnosis. However, it should be noted that the distribution of health conditions across the diagnostic profiles of the CSHCN categories validates our approach to creating our own discrete categories among CSHCN (eTable 1 in Supplement 1). Second, measures of health insurance adequacy were based on parents’ self-reported perceptions rather than objective characteristics. We acknowledge that perceptions of reasonable health care costs and out-of-pocket expenses vary across income levels. The perception of insurance adequacy (outcome variable) may also vary by level of health care needs (exposure variable). Third, this national-level study does not account for complex state-level variation in income- and condition-based eligibility for Medicaid and CHIP that may be associated with differential experiences of underinsurance across state lines.

## Conclusions

In this cross-sectional study, we found that during a period of rising out-of-pocket costs for many families enrolled in commercial insurance, the likelihood of underinsurance among CSHCN increased with the severity of children’s health care needs. Underinsurance was more prevalent among CSHCN with complex physical conditions and limitations—particularly so for middle-income households—as well as among children with mental or behavioral conditions and limitations who have unique and challenging health care needs. As health care and insurance costs continue to rise, ongoing evaluation will be necessary so that state and federal health authorities can modify public health insurance program design and eligibility to meet the needs of CSHCN best.

## Notes

Supplement 1. **eTable 1.** Description and Composition of Child Health Needs Categories Created Using Questions From NSCH 2016 to 2021 **eAppendix. **Control Variables **eTable 2. **Characteristics of Children in the United States, by Children's Healthcare Needs Category, National Survey of Children's Health (NSCH) (2016 to 2021) **eTable 3.** Risk Ratios for Underinsurance Among Children in the United States, NSCH 2016 to 2021

Supplement 2.
Data Sharing Statement
