# Supplementary material for: Underinsurance Among Children With Special Health Care Needs in the United States
Source: JAMA Netw Open. 2023 Dec 26;6(12):e2348890. doi: 10.1001/jamanetworkopen.2023.48890 (PMC10751585; doi:10.1001/jamanetworkopen.2023.48890)
Supplement: Supplement 1. — eTable 1. Description and Composition of Child Health Needs Categories Created Using Questions From NSCH 2016 to 2021 eAppendix. Control Variables eTable 2. Characteristics of Children in the United States, by Children's Healthcare Needs Category, National Survey of Children's Health (NSCH) (2016 to 2021) eTable 3. Risk Ratios for Underinsurance Among Children in the United States, NSCH 2016 to 2021 [file jamanetwopen-e2348890-s001.pdf]

Supplemental Online Content

Validova A, Strane D, Matone M, et al. Underinsurance among children with special healthcare needs in the United States *JAMA Netw Open*. 6(12):e2348890. doi:10.1001/jamanetworkopen.2023.48890

eTable 1. Description and Composition of Child Health Needs Categories Created Using Questions From NSCH 2016 to 2021

eAppendix. Control Variables

eTable 2. Characteristics of Children in the United States, by Children's Healthcare Needs Category, National Survey of Children's Health (NSCH) (2016 to 2021)

eTable 3. Risk Ratios for Underinsurance Among Children in the United States, NSCH 2016 to 2021

This supplemental material has been provided by the authors to give readers additional information about their work.

© 2023 Validova A et al. *JAMA Network Open.*

eTable 1. Description and composition of child health needs categories created using questions from NSCH 2016 to 2021

Child health status category	Description	NSCH questions					Diagnosis profile (most prevalent conditions among health care needs category)
		Q1	Q2	Q3	Q4	Q5	
		Does this child CURRENTLY need or use medicine prescribed by a doctor, other than vitamins?	Does this child need or use more medical care, mental health, or educational services than is usual for most children of the same age?	Does this child need or get special therapy, such as physical, occupational, or speech therapy?	Does this child have any kind of emotional, developmental, or behavioral problem for which he or she needs treatment or counseling?	Is this child limited or prevented in any way in their ability to do the things most children of the same age can do?	
Healthy children	Children with no notable illnesses or limitations	No	No	No	No	No	

© 2023 Validova A et al. *JAMA Network Open.*

Children with ongoing medication needs	Children with chronic health conditions that require the use of prescribed medication	Yes	No	No	No	No	Asthma (42.2%)<br>Breathing or respiratory problems (28.5%)<br>ADHD (21.0%)<br>Anxiety (11.7%)<br>Chronic physical pain (10.4%)
Children with complex physical health conditions but no limitations	Children with chronic health conditions that require special therapy or need/use more medical care than others but do not have functional limitations	Yes/no	Yes	Yes	No	No	Asthma (26.8%)<br>Breathing or respiratory problems (21.5%)<br>ADHD (21.0%)<br>Speech disorder (20.0%)<br>Developmental delay (14.8%)<br>Learning disabilities (14.5%)<br>Anxiety (14.2%)<br>Chronic physical pain (13.4%)<br>Chronic stomach, intestinal problems (13.4%)<br>Heart condition (7.7%)

© 2023 Validova A et al. *JAMA Network Open.*

Children with mental/behavioral health conditions but no limitations	Children with chronic health conditions that require treatment of emotional, developmental, or behavioral problems but do not have functional limitations	Yes/no	Yes/no	Yes/no	Yes	No	Anxiety (56.9%)<br>ADHD (49.6%)<br>Behavioral problems (47.3%)<br>Depression (34.8%)<br>Learning disability (21.8%)
Children with complex physical health conditions and limitations	Children with chronic health conditions that require special therapy or need/use more medical care than others and have functional limitations	Yes/no	Yes	Yes	No	Yes	Developmental delay (37.8%)<br>Speech disorder (33.3%)<br>Learning disability (29.5%)<br>Asthma (27.0%)<br>Chronic stomach, intestinal problems (25.5%)<br>Breathing or respiratory problems (25.2%)<br>ADHD (15.7%)<br>Chronic physical pain (15.5%)

© 2023 Validova A et al. *JAMA Network Open.*

							Anxiety (14.9%)<br>Heart condition (12.5%)<br>ASD (10.5%)<br>Deafness (9.4%)
Children with mental/behavioral health conditions and limitations	Children with chronic health conditions that require treatment of emotional, developmental, or behavioral problems and have functional limitations	Yes/no	Yes/no	Yes/no	Yes	Yes	Developmental delay (67.7%)<br>Learning disability (60.5%)<br>Behavioral problems (59.9%)<br>Anxiety (53.7%)<br>Speech disorder (52.4%)<br>ADHD (48.9%)<br>ASD (41.3%)

Notes:

1) Chronic health conditions are defined as lasting 12 months or more.
2) Each survey question was followed by additional questions that specified if the child needed the prescribed medication/more medical care than others/special therapy/treatment for emotional/behavioral problem or had limited ability because of any medical, behavioral, or other health condition that lasted or is expected to last 12 months or longer. A positive answer means answering "yes" to all the questions including clarifying questions.
3) To be categorized as a child with complex physical health conditions but no limitations the respondent had to answer positively to Q2 or Q3.
4) To be categorized as a child with complex physical health conditions and limitations the respondent had to answer positively to Q2 or Q3, and Q5.

© 2023 Validova A et al. *JAMA Network Open.*

eAppendix.

Control Variables

We included the following demographic control variables: age, sex, race/ethnicity and child immigrant status. Age was a categorical variable and included three age groups: 0-5, 6-11 and 12-17-year-old children. Race/ethnicity variable distinguished between four categories: Non-Hispanic Whites (reference category), Non-Hispanic Blacks, Hispanics, and Other racial/ethnic group. The questions about child's Hispanic origin and race were asked in the initial screening questionnaire. Non-Hispanic children reporting one race category of Asian, American Indian or Alaska Native, Native Hawaiian or other Pacific Islander, and multi-race were grouped as other. Following NSCH classification of child's immigrant status, we distinguished between 1st generation household (child is born outside of the United States), 2nd generation household (child is born in the United States and at least one parent is born outside the U.S.), 3rd+ generation household (both parents are born in the U.S.) and other (child is born in the U.S., parents are not listed).

The following socio-economic variables were included in the analysis: parental educational attainment, household income, and type of health insurance coverage. Highest parental education was categorized as "less than high school", "high school", "some college or associate degree", and "college degree or higher". Household income variable (measured as a percentage of the federal poverty level (FPL)) was derived by NSCH from responses on the survey about household income and was generated using single imputation method. Four categories were created: 0-99% FPL (reference category), 100-199% FPL, 200-399% FPL, and 400% FPL or above. Finally, previously shown to significantly predict underinsurance (Yu et al, 2022), type of health insurance coverage was included in our models. Insurance type was categorized as follows: (a) only public health insurance, defined as Medicaid, Medical Assistance, or any kind of government assistance plan with those with low income or a disability, (b) only private insurance, (c) combined public and private health insurance, and (d) no current insurance coverage.

© 2023 Validova A et al. *JAMA Network Open.*

eTable 2. Characteristics of Children in the United States, by Children's Healthcare Needs Category, National Survey of Children's Health (NSCH) (2016 to 2021)

	Healthy children		Children with ongoing medication needs		Children with complex physical conditions but no limitations		Children with mental/behavioral conditions but no limitations		Children with complex physical conditions and limitations		Children with mental/behavioral conditions and limitations	
**N (unweighted)**	**N=170,869**		**N=13,654**		**N=8,025**		**N=14,265**		**N=3,091**		**N=8,022**	
	N	*Row%*	N	*Row %*	N	*Row %*	N	*Row %*	N	*Row %*	N	*Row %*
Dependent variable												
**Underinsurance***												
Insured	118,440	*83*	9,249	*4.8*	4,709	*2.8*	8,833	*4.9*	1,684	*1.1*	4,580	*2.9*
Underinsured	52,429	*80*	4,405	*4.8*	3,316	*3.5*	5,432	*6.4*	1,407	*1.6*	3,442	*4.3*
Independent variables												
**Age group**												
0-5 years	60,893	*90*	2,157	*2.5*	2,003	*2.4*	1,072	*1.5*	767	*1.1*	1,499	*2.1*
6-11 years	49,264	*80*	4,174	*5.1*	2,697	*3.3*	5,135	*6.3*	996	*1.3*	2,750	*4.0*
12-17 years	61,290	*76*	7,348	*6.7*	3,348	*3.2*	8,100	*8.2*	1,337	*1.5*	3,791	*3.9*
**Sex**												
Female	85,125	*80*	6,231	*5.1*	3,502	*3.2*	6,543	*5.9*	1,296	*1.5*	2,781	*4.4*
Male	86,322	*85*	7,448	*4.5*	4,546	*2.8*	7,764	*4.8*	1,804	*1.0*	5,259	*2.3*
**Race/ethnicity**												

© 2023 Validova A et al. *JAMA Network Open.*

Hispanic	21,301	*84*	1,382	*3.7*	903	*2.7*	1,650	*4.5*	397	*1.4*	1,013	*3.3*
NH Black	10,130	*78*	1,059	*6.1*	527	*3.1*	980	*6.6*	212	*1.5*	663	*4.8*
NH White	116,749	*82*	9,699	*5.1*	5,675	*3.2*	10,118	*5.8*	2,129	*1.2*	5,336	*3.1*
Other[1]	23,267	*85*	1,539	*4.4*	943	*2.8*	1,559	*4.0*	362	*1.2*	1,028	*3.0*
**Nativity**												
1st generation immigrants	3,271	*90*	106	*2.4*	59	*1.6*	103	*3.6*	35	*0.6*	78	*1.9*
2nd generation immigrants	29,072	*88*	1,541	*3.2*	955	*2.4*	1,346	*3.1*	370	*1.0*	962	*2.6*
3rd + generation	131,435	*80*	11,359	*5.5*	6,690	*3.3*	11,518	*5.9*	2,534	*1.4*	6,370	*3.5*
Other/unknown	7,669	*78*	673	*4.6*	344	*2.4*	1,340	*9.0*	161	*1.8*	630	*4.6*
**Household income (% FPL)**												
0-99%	18,659	*80*	1,395	*4.6*	852	*2.8*	2,029	*6.4*	437	*1.5*	1,453	*4.8*
100-199%	26,842	*81*	2,059	*4.5*	1,312	*2.9*	2,572	*5.5*	606	*1.7*	1,680	*4.0*
200-399%	53,202	*83*	4,123	*4.8*	2,423	*3.0*	4,270	*5.1*	998	*1.1*	2,367	*2.9*
400% FPL or greater	72,744	*83*	6,102	*5.1*	3,461	*3.3*	5,436	*4.9*	1,059	*1.1*	2,540	*2.4*
**Parental education**												
Less than high school	4,218	*86*	227	*2.8*	132	*2.1*	302	*4.9*	79	*1.2*	196	*3.3*
High school	21,661	*82*	1,601	*4.8*	914	*2.7*	1,985	*5.5*	418	*1.4*	1,262	*4.0*
Some college	37,457	*80*	3,164	*5.2*	1,807	*3.1*	3,727	*6.5*	799	*1.5*	2,268	*4.2*
College or more	108,111	*83*	8,687	*5.0*	5,195	*3.3*	8,293	*5.0*	1,804	*1.2*	4,314	*2.8*

© 2023 Validova A et al. *JAMA Network Open.*

Type of health insurance coverage												
Public only	32,047	*78*	2,688	*4.7*	1,693	*3.2*	4,388	*7.3*	876	*1.6*	3,003	*5.3*
Private only	126,037	*85*	10,148	*5.1*	5,678	*3.0*	8,513	*4.4*	1,777	*1.0*	3,491	*1.9*
Private and public	5,001	*69*	430	*4.8*	417	*3.7*	927	*8.3*	334	*3.1*	1,285	*11*
Not insured	7,968	*89*	385	*2.6*	241	*1.7*	454	*3.5*	106	*1.0*	247	*1.7*

Note:
1) N is the number of observations (unweighted), % is calculated adjusting for weights and survey design
2) *Underinsurance is defined as failure to meet either of the following: 1) continuous insurance in the past 12 months, and 2) current insurance that is adequate for the child's healthcare needs. Health insurance is defined as adequate if: (a) benefits meet child's needs, and (b) insurance allows the child to see needed providers, and (c) the insurance either has no out-of-pocket expenses or out-of-pocket expenses are reasonable.
3) [1]"Other" racial/ethnic category included Non-Hispanic children reporting one race category of Asian, American Indian or Alaska Native, Native Hawaiian or other Pacific Islander, and children who reported more than one race.

eTable 3. Risk ratios* for underinsurance among children in the United States, NSCH 2016-2021

	Model 1[1] N=217,926		Model 2 [2] N=217,926		Model 3[3] N=217,926	
	**RR**	**95% CI**	**RR**	**95% CI**	**RR**	**95 % CI**
**Children's healthcare needs category (reference: healthy children)**						
Children with ongoing medication needs	1.00	[0.99, 1.02]	1.00	[0.99, 1.02]	0.98	[0.95, 1.02]
Children with complex physical conditions but no limitations	1.04	[1.03, 1.06]	1.05	[1.03, 1.06]	1.01	[0.96, 1.06]

© 2023 Validova A et al. *JAMA Network Open.*

Children with mental/behavioral conditions but no limitations	1.05	[1.04, 1.07]	1.05	[1.04, 1.07]	1.01	[0.97, 1.05]
Children with complex physical conditions and limitations	1.07	[1.04, 1.11]	1.08	[1.04, 1.11]	1.00	[0.94, 1.07]
Children with mental/behavioral conditions and limitations	1.07	[1.06, 1.09]	1.08	[1.06, 1.10]	1.02	[0.98, 1.06]
**Demographic controls**						
**Sex (reference: male)**						
Female			1.01	[1.00, 1.02]	1.01	[1.00, 1.02]
**Age group (reference: 0-5)**						
6-11			1.02	[1.01, 1.03]	1.02	[1.01, 1.03]
12-17			1.04	[1.03, 1.05]	1.04	[1.03, 1.05]
**Race/ethnicity (reference: NH White)**						
Hispanic			1.01	[1.00, 1.02]	1.01	[1.00, 1.02]
NH Black			0.97	[0.96, 0.98]	0.97	[0.96, 0.98]
Other[4]			0.99	[0.97, 1.00]	0.99	[0.98, 1.00]
**Nativity (reference: 3rd + generation)**						
1st generation immigrants			1.12	[1.09, 1.14]	1.12	[1.09, 1.14]
2nd generation immigrants			1.01	[1.00, 1.02]	1.01	[1.00, 1.02]
Other			0.97	[0.95, 0.98]	0.97	[0.95, 0.98]

© 2023 Validova A et al. *JAMA Network Open.*

<u>**Socioeconomic variables**</u>				
**Parental education (reference: less than high school)**				
High school	0.95	[0.93, 0.97]	0.95	[0.93, 0.97]
Some college	0.96	[0.94,0.98]	0.96	[0.94, 0.98]
College or higher	0.96	[0.95, 0.98]	0.96	[0.95, 0.98]
**Parental income (reference: 0-99% FPL)**				
100-199% FPL	1.02	[1.00, 1.03]	1.01	[1.00, 1.03]
200-399 % FPL	1.07	[1.06, 1.08]	1.06	[1.04, 1.07]
400 FPL or greater	1.03	[1.02, 1.04]	1.01	[1.00, 1.03]
**Year (reference: 2016)**				
2017	1.02	[1.00, 1.03]	1.02	[1.00, 1.03]
2018	1.01	[1.00, 1.02]	1.01	[1.00, 1.02]
2019	1.03	[1.01, 1.04]	1.03	[1.01, 1.04]
2020	1.01	[1.00, 1.02]	1.01	[1.00,1.02]
2021	1.00	[0.99, 1.01]	1.00	[0.99, 1.01]
**Child healthcare needs category*parental income**				
Children with ongoing medication needs *100-199% FPL			1.04	[0.99, 1.09]

© 2023 Validova A et al. *JAMA Network Open.*

Children with ongoing medication needs *200-399% FPL	1.02	[0.97, 1.06]
Children with ongoing medication needs *400% FPL or greater	1.03	[0.99, 1.07]
Children with complex physical conditions but no limitations *100-199% FPL	1.00	[0.94, 1.07]
Children with complex physical conditions but no limitations *200-399% FPL	1.04	[0.98, 1.10]
Children with complex physical conditions but no limitations *400% FPL or greater	1.08	[1.01, 1.14]
Children with mental/behavioral conditions but no limitations *100-199% FPL	1.01	[0.96, 1.06]
Children with mental/behavioral conditions but no limitations *200-399% FPL	1.05	[1.00, 1.09]
Children with mental/behavioral conditions but no limitations *400% FPL or greater	1.09	[1.04, 1.13]
Children with complex physical conditions and limitations *100-199% FPL	1.03	[0.93, 1.15]
Children with complex physical conditions and limitations *200-399% FPL	1.14	[1.05, 1.23]
Children with complex physical conditions and limitations *400% FPL or greater	1.12	[1.03, 1.22]

© 2023 Validova A et al. *JAMA Network Open.*

Children with mental/behavioral conditions and limitations *100-199% FPL					1.04	[0.98, 1.10]
Children with mental/behavioral conditions and limitations *200-399% FPL					1.06	[1.00, 1.11]
Children with mental/behavioral conditions and limitations*400% FPL or greater					1.17	[1.11, 1.23]
**Constant**	1.31	[1.30, 1.32]	1.27	[1.24, 1.30]	1.28	[1.25, 1.31]

Notes:

1) * Modified Poisson regression was used to generate RR and CI

2) [1] Model 1 is unadjusted model

[2] Model 2 has been adjusted for demographic and socioeconomic variables

[3]Model 3 is an adjusted model which includes interaction between child healthcare needs and household income level

3) [4]"Other" racial/ethnic category included Non-Hispanic children reporting one race category of Asian, American Indian or Alaska Native, Native Hawaiian or other Pacific Islander, and children who reported more than one race.

© 2023 Validova A et al. *JAMA Network Open.*
