# Supplementary material for: Underinsurance Among Children With Special Health Care Needs in the United States
Source: JAMA Netw Open. 2023 Dec 26;6(12):e2348890. doi: 10.1001/jamanetworkopen.2023.48890 (PMC10751585; doi:10.1001/jamanetworkopen.2023.48890)
Supplement: Supplement 2. — Data Sharing Statement [file jamanetwopen-e2348890-s002.pdf]

Data Sharing Statement

Validova. Underinsurance Among Children With Special Health Care Needs in the United States. *JAMA Netw Open*. Published December 26, 2023.
doi:10.1001/jamanetworkopen.2023.48890

Data
Data available: Yes
Data types: Other (please specify)
Additional Information: For this research NSCH public-use files (PUF) were used which are available on the Census Bureau's NSCH page: https://www.childhealthdata.org/learn-about-the-nsch/NSCH
How to access data: For this research NSCH public-use files (PUF) were used which are available on the Census Bureau's NSCH page: https://www.childhealthdata.org/learn-about-the-nsch/NSCH
When available: With publication

Supporting Documents
Document types: None

Additional Information
Who can access the data: Anyone requesting the data
Types of analyses: For any purpose
Mechanisms of data availability: Publicly used data
